# Quality Chemistry, Physiological Functions, and Health Benefits of Organic Acids from Tea (*Camellia sinensis*)

**DOI:** 10.3390/molecules28052339

**Published:** 2023-03-03

**Authors:** Hongbo Chen, Fei Yu, Jiaxin Kang, Qiao Li, Hasitha Kalhari Warusawitharana, Bo Li

**Affiliations:** 1 Modern Service Industry Research Institute, Zhejiang Shuren University, Hangzhou 310015, China; 2 Department of Tea Science, Zhejiang University, Hangzhou 310058, China

**Keywords:** tea, organic acids, physiological function, sensory quality, health benefit

## Abstract

Organic acids account for around 3% of the dry matter in tea leaves, and their composition and contents vary in different types of tea. They participate in the metabolism of tea plants, regulate nutrient absorption and growth, and contribute to the aroma and taste quality of tea. Compared with other secondary metabolites in tea, the researches on organic acids are still limited. This article reviewed the research progresses of organic acids in tea, including analysis methods, the root secretion and physiological function, the composition of organic acids in tea leaves and related influencing factors, the contribution of organic acids to sensory quality, and the health benefits, such as antioxidation, promotion of digestion and absorption, acceleration of gastrointestinal transit, and regulation of intestinal flora. It is hoped to provide references for related research on organic acids from tea.

## 1. Introduction

Organic acids are a class of organic compounds with acidity that originates from the carboxyl group (-COOH), sulfonic group (-SO_3_H), sulfinic group (-RSOOH), and carboxylic group (-RCOSH), etc. Carboxylic acids are the most common type of organic acids. They are divided into aliphatic, aromatic, and terpenoid compounds based on their structures and into monocarboxylic acids, dicarboxylic acids, and polycarboxylic acids according to the number of carboxylic groups [1]. Organic acids are the intermediate products of carbohydrates in biochemical reactions and are the participants in plant metabolic activities. They are also important intermediates in metabolic pathways, such as the tricarboxylic acid cycle and shikimic acid pathway, and play a vital role in energy transfer and maintenance of cell osmotic pressure [2,3]. Organic acids in tea are mainly aliphatic acids (such as oxalic acid, malic acid, and citric acid) and aromatic acids (such as benzoic acid, salicylic acid, and cinnamic acid). Until now, more than 40 organic acids have been discovered in different tea, including more than 10 in tea soup and over 30 in its aroma. Organic acids were thought to contribute to the tea flavor, epically the sourness of tea soup, and have a variety of health benefits for the human body, such as regulation of intestinal flora and antioxidation. In addition, organic acids influence nutrient absorption of tea plants, such as phosphorus and aluminum ions. Compared with other metabolites of tea plants, such as catechin, theine, theanine, and flavonoid glycosides, there are few studies on organic acids in tea [4]. In this paper, the detection method of organic acids, their composition and function in tea roots and leaves, the influence of the plant variety and processing technology on them, as well as their health functions, are reviewed to provide references for future studies.

## 2. Detection Method of Organic Acids

The titration method, atomic absorption method, gas chromatography, ion exchange chromatography, high-performance capillary electrophoresis, and high-performance liquid chromatography are the main methods to detect the organic acids in tea. He et al. [5] introduced the principle, application, advantages, and disadvantages of the above detection methods, according to which this paper makes a further summary, as shown in Table 1. The most commonly used method for the analysis of organic acids is high-performance liquid chromatography (HPLC), which mostly uses the C_18_ column as the chromatographic column and tends to add phosphoric acid to the mobile phase. Phosphoric acid is more acidic than formic acid and acetic acid, which are commonly used in high-performance liquid chromatography mobile phase. Plus, it has no UV background or volatility with moderate ionic strength. However, owing to the generally small molecular weight and high polarity of organic acids, the separation degree of organic acids on HPLC is usually low. In addition, the phosphate in the mobile phase is prone to crystallize and tends to block the instrument pipeline, causing damage to instruments and thus increasing the cost of equipment operation and maintenance [6,7,8,9,10].

With the development of modern analytical technology, more and more researchers use ultra-high liquid chromatography-mass spectrometry (UPLC-MS) technology to detect organic acids and quantitative analysis carried out under multi-reaction detection mode (MRM) [11,12,13,14,15,16,17,18]. The mobile phase of this method does not need to use phosphoric acid, but mainly uses formic acid or acetic acid, water and acetonitrile. The method also enjoys exquisite specificity, avoiding the qualitative and quantitative interference caused by the close peak time and low separation degree between different organic acids. Plus, the sensitivity and accuracy of the method are also improved. Nowadays, this technique has been used for the detection of organic acids in fruits, tobacco leaves, and Chinese medicinal herbs. The author summarizes the MRM detection conditions of common organic acids in tea, such as fragment ion pair, cone hole voltage, and collision pressure [17,18,19] (Table 2), all using electrospray ionization (ESI) and negative ion mode. In this way, the article hopes to provide a reference for the establishment of the method for the detection of organic acids in tea leaves.

## 3. Secretion and Function of Organic Acids in Tea Roots

The types and contents of organic acids secreted by the root system of tea plants are significantly different. The influencing factors mainly include varieties, seasons, light conditions, soil pH, etc. The organic acids secreted by roots are of great significance to the absorption of nutrients and the normal growth of tea plants.

### 3.1. Types and Contents of Organic Acids in Tea Roots

The organic acids secreted by the root system of the tea plant mainly include tartaric acid, oxalic acid, malic acid, citric acid, and succinic acid [20,21,22]. The types and contents of organic acids secreted by roots vary in different seasons and varieties. For the tea variety Longjing 43, it was found that in spring, citric acid is secreted the most of all the organic acids (1007 μg/individual plant), and oxalic acid and malic acid are the least. In summer, succinic acid is secreted the most (1500 μg/individual plant), and oxalic acid is the least. In autumn, the secretion of oxalic acid is the highest (388 μg/individual plant), and the secretion of citric acid is the lowest. In terms of the total amount of oxalic acid, succinic acid, malic acid, and citric acid, the secretion is the highest in summer (1856 μg/individual plant) and least in autumn (444 μg/individual plant). The three different tea varieties, Biyun, Fuding, and Longjing 43, have different total organic acid secretion in autumn, with Fuding the most (1213 μg/individual plant) and Biyun the least (50 μg/individual plant). Light is essential for the secretion of organic acids. As an intermediate product of metabolism, organic acids are mostly produced in the process of the tricarboxylic acid cycle, and their secretion is inseparable from the photosynthesis of plants. Under light conditions, the total amount of organic acids secreted by the roots of Longjing 43 and the Fuding tea tree is much higher than that under dark conditions, while oxalic acid is scarcely secreted under dark conditions [21].

Pang et al. found that tea polyphenols could induce the secretion of malic acid and citric acid from the root system of the tea plant. Additionally, a proper amount of water-soluble material trimmings can induce the secretion of oxalic acid, malic acid, citric acid, and succinic acid, thus reducing the pH value of the collected solution. Therefore, the water-soluble components of tea plant trimming may be one of the causes of soil acidification in tea plantations. Soil pH has a major effect on the secretion of organic acids from tea plants [22]. Tang et al. took Longjing 43 as the material and found that the concentration of oxalic acid in roots and that of malic acid, citric acid, and oxalic acid in mature leaves are increased at pH 6.0 relative to the condition of pH 4.0 and pH 5.0 [23].

### 3.2. Organic Acids in Tea Roots and Nutrient Absorption

The organic acids secreted by the root system of tea tree can improve tea plants’ absorption of phosphorus elements, reduce the adsorption of fluorine by the iron film on the root surface of tea plants and play a significant role in the healthy growth of tea plants in a high-aluminum acid environment. The organic acids secreted by tea plants during their growth process can dissolve insoluble inorganic phosphorus in the soil. After organic acid treatment, the content of insoluble inorganic phosphorus in soil decreases to varying degrees. Among them, oxalic acid has the strongest effect, followed by citric acid and malic acid, and succinic acid has the weakest solubility [21]. The secretion of organic acids from roots may be one of the main reasons for the strong ability of tea plants to absorb phosphorus. Tea is a typical plant with high enrichment of fluorine elements, especially in crude and old tea leaves. The fluorine element is mainly ingested from the roots of the tea plant and accumulated in the leaves [24,25]. The content of Fe^2+^ and Al^3+^ in the soil of a tea garden is high, and thus the iron oxide film is easy to form on the root surface of a tea tree, which will promote the absorption of fluorine by a tea tree. With the increase of organic acid concentration, the adsorption capacity of root surface iron film on fluorine decreases [26]. Liu et al. conducted hydroponic experiments on tea seed seedlings of the Fuding white tea variety and found that under the stimulation of aluminum ions, the roots mainly secreted oxalic acid, malic acid, and citric acid, accounting for 85–93% of the total amount of organic acid secreted. The secretion of oxalic acid would increase with the concentration of aluminum ions rose. Therefore, oxalic acid may be able to balance the concentration of aluminum ions in tea tree roots. Given the fact that tea trees can still grow well in a high-aluminum acidic environment, it is likely that organic acids play an active role in alleviating aluminum toxicity [27,28].

Organic acids in the root system of tea trees are vital to the material circulation in the soil of tea gardens. Lin et al. chose Fujian Jiukeng seedlings as experimental materials and found that under the environment of Pb^2+^ and Cd^2+^, the composition of organic acids secreted by roots did not change, mainly including oxalic acid, malic acid, lactic acid, acetic acid, citric acid, and succinic acid. However, the amount of each acid changes to varying degrees. Among them, the secretion of succinic acid and malic acid increases significantly, while citric acid decreases noticeably. This may be an important mechanism for the complex detoxification of organic acids and metal ions [29]. Zeng et al. found that organic acids such as citric acid, malic acid, tartaric acid, and oxalic acid can activate F, P, Zn, Fe, Al, Cu, and Mn elements in the soil [30].

## 4. Organic Acids in Tea

The composition and content of organic acids in tea leaves are closely related to the type, processing technology, variety, and growth period of tea [7,31,32,33,34,35,36].

Tukhvatshin et al. showed that the organic acids in Fu’an white tea leaves are mainly citric acid, gallic acid, benzoic acid, oxalic acid, salicylic acid, ascorbic acid, and chlorogenic acid. Among them, the content of citric acid was the highest, p-coumaric acid and fumaric acid were the lowest, and succinic acid was not detected. The total amount of organic acids in white tea processing ranged from 5.6839 to 10.1014 mg/g, and the order is crude tea > 24H > 48H > 32H > 40H > 16H > 8H > fresh leaf. The contents of citric acid, gallic acid, oxalic acid, caffeic acid, cinnamic acid, and acetic acid show an increasing trend. Among them, the contents of citric acid, gallic acid, and oxalic acid in crude white tea are 4.7873, 2.4122, and 1.2758 mg/g, which are respectively 2.9, 1.4, and 1.7 times those in fresh leaves. During the processing of white tea, the contents of chlorogenic acid and p-coumaric acid show a decreasing trend. The contents of chlorogenic acid and p-coumaric acid in fresh leaves are 0.1614 and 0.0366 mg/g, 1.6 and 3.0 times those of white tea, respectively. There is no obvious change in the content of benzoic acid, salicylic acid, ferulic acid, fumaric acid, and ascorbic acid content. The content of benzoic acid peaked eight hours after withering, ascorbic acid 16 h, salicylic acid 24 h, and ferulic acid 48 h [31]. He et al. conducted an analysis of the components of organic acids in Silver Needle Pekoe, White Peony, and Shou-mee and found that with the decrease of leaf freshness, the number of organic acids in white tea decreased, among which the content of lactic acid, acetic acid, citric acid, and fumaric acid gradually decreases. However, the content of malic acid increases with the loss of freshness, while oxalic acid and ascorbic acid have no distinct change pattern. Overall, acetic acid is the main organic acid in the three kinds of white tea samples, accounting for 32.8%, 46.5%, and 42.5% of the total organic acid [6].

During black tea processing, the total amount of organic acids, including oxalic acid, citric acid, succinic acid, malic acid, etc., significantly increases after withering, rolling, and fermentation. The contents of organic acids are 1.44%, 1.51%, and 1.72% in fresh leaves, withered leaves, and rolled leaves, respectively. After drying, the organic acid content in the tea leaves will decrease [35]. Yu et al. explored dynamic changes of organic acids during black tea processing by a UPLC-MRM-MS method. The results showed that the contents of quinic acid and citric acid increased and then decreased during the withering process, but there was no obvious change during the whole process. Malic acid content obviously decreased at the early stage of withering, and gallic acid content markedly increased after rolling [37]. Xie et al. found that the total amount of organic acids in Keemun black tea stored at room temperature for 6 years considerably increased, but the tendency of each organic acid compound was different. For example, the content of oxalic acid and acetic acid greatly increased, while the citric acid significantly decreased, leading to the overall sour and poor taste of tea soup [33]. Mao analyzed 32 Gongfu black teas produced in 14 provinces and used HPLC to determine eight organic acids, including oxalic acid, pyruvic acid, L-malic acid, L-ascorbic acid, lactic acid, acetic acid, citric acid, and succinic acid. It was found that the content of L-malic acid in Gongfu black tea samples was the highest, followed by oxalic acid and succinic acid, and the content of acetic acid was the lowest, which was only detected in some samples. The content of L-malic acid and acetic acid in different types of Gongfu black teas greatly varies [38]. Zhang et al. analyzed six black teas from different origins, including Ceylon black tea from Sri Lanka, Ninghong from Jiangxi, Dianhong from Yunnan, Yinghong from Guangdong, Keemun black tea from Anhui and Shimen black tea from Hunan. A total of 11 organic acids are detected: oxalic acid, tartaric acid, formic acid, pyruvic acid, malic acid, ascorbic acid, lactic acid, acetic acid, citric acid, succinic acid, and fumaric acid, among which oxalic acid and acetic acid are the main organic acids [10].

Li et al. detected nine organic acids, namely oxalic acid, tartaric acid, pyruvate, malic acid, acetic acid, citric acid, succinic acid, fumaric acid, and α-ketoglutaric acid, during the flowering process of dark tea. It is found that the amount of malic acid decreases by 85.8%, while the content of succinic acid increases by 8.42 times that of before fermentation [39]. Ding found that the total amount of organic acids in Fuzhuan brick tea processing increased from 42.63 mg/g in raw tea to 43.44 mg/g in the finished tea. After 5 days of flowering, the content of D-malic acid and α-ketoglutarate acid decreases, while ascorbic acid, acetic acid, and citric acid increase [40].

Liu studied the content of organic acids in different leaf positions of one-bud four-leaf Cuifeng species, Longjing 43, Jiukeng, Huangjingui, Yunkang 10, and Fuding 5, as well as representative teas on sale, such as black tea, green tea, oolong tea, and Pu ‘er tea. He also studied the leaching characteristics of organic acids in Wuyi green tea under different brewing conditions. In terms of the leaf position, the total amount of organic acid decreases with the decrease in fresh leaf tenderness. The content of oxalic acid decreases significantly, and the content of organic acids in the fourth leaf is only 49.5% of that in one-bud one-leaf. After comparing the content of organic acids in five different varieties of processed, roasted green tea, it is found that the content of organic acids in Fuding species is the lowest (0.538 mg/mL), the content of Huangjingui is the highest (0.716 mg/mL), and the content of oxalic acid, quinic acid, and L-ascorbic acid in Yunkang 10 of large leaf species is higher than those of medium and small leaf species. The content of organic acids in different kinds of tea significantly changes, and the order is black tea > oolong tea > green tea > Pu ‘er tea. Quinic acid, L-ascorbic acid, and citric acid are the main organic acids leading to the difference. The processing technology, especially the degree of fermentation, is positively correlated with the total content of organic acids. The lowest content of Pu ‘er tea may be related to the tenderness of raw materials, post-fermentation process, and storage time. In addition, the leaching of organic acids rises with the increase in brewing temperature and brewing time but decreases with the increase in pH value [32,36].

Zhang et al. treated the Longjing 43 variety with a sunshade net and straws for 20 days and found that the content of organic acids in leaves and stems changed differently. Compared with the control group, the relative contents of succinic acid and quinic acid in leaves decreased, and malic acid, gallic acid, citric acid, and α-ketoglutarate increased. In stems, quinic acid decreases under sunshade net cover and increases under straw cover [41]. Shirai found that the content of quinic acid was high in Kukicha and low in matcha. The quinic acid content increased with leaf maturity, as opposed to oxalic, malic, succinic, and citric acids. Shading cultivation markedly reduced the quinic acid content and slightly enhanced the content of malic, citric, and oxalic acids. Low-grade green tea is a good source of quinic acid [42].

## 5. Organic Acids and Sensory Quality of Tea

Although organic acids only account for about 3% of the dry matter weight, it has a great impact on the quality of tea leaves. Aromatic organic acids and aliphatic organic acids in tea leaves contribute a lot to the formation of tea aroma and taste. Most of these organic acids are soluble in water and are one of the main ingredients affecting the flavor and quality of tea soup [4].

### 5.1. Organic Acids and Tea Aroma

The content of carboxylic acid in fresh tea leaves is lower than that in the finished tea. One of the reasons for the flavor difference between black tea and green tea is the different content and proportion of volatile organic acids. Studies have shown that the organic acids account for about 30% of the total essential oils in black tea and only account for 2–3% in green tea. During the withering and fermentation process of black tea, unsaturated fatty acids will be oxidized and degraded into volatile small molecules, such as aldehydes, ketones, and acids, which then participate in the composition of black tea aroma [43]. During the processing of Keemun black tea, the content of most carboxylic acid aromatic substances gradually increases, especially aromatic substances, such as cinnamic acid, which constitutes the special aroma of Keemun black tea [35]. During the storage process of tea leaves, the free fatty acids produced from the hydrolysis of lipid compounds are the main reason for the musty and rancid tastes [44]. Under extreme environments, such as high-temperature storage conditions, aerobic environments, and intense light, lipids in tea leaves will be hydrolyzed or oxidized, resulting in the deterioration or aging of tea leaves [45]. During the storage process of green tea, the content of organic acids such as oleic acid and stearic acid, which are negatively correlated with the quality of tea aroma, will increase. However, the content of organic acids, such as palmitic acid, lauric acid, and linolenic acid, which are positively related to the quality of tea aroma, will decrease [46].

### 5.2. Organic Acids and Tea Flavor

The organic acids are one class of main components contributing to the sour taste of tea soup, whose content is positively correlated with the degree of fermentation. Moderate content of sour compounds increases the fullness of taste, but excessive content will lead to the deterioration of quality [36,47]. Yue et al. found that sour taste was related to malic acid, lactic acid, citric acid, and shikimic acid through the partial least squares and variable projection analysis [48]. However, Zhang et al. reported that the acidity in black tea and green tea was not directly related to organic acids but associated with the interaction between organic acids and other substances, pH value, or other sour substances in tea infusions [49].

The taste threshold is the critical concentration value at which each taste compound can be perceived. The lower it is, the easier it is to be perceived. The taste activity value is the ratio of the content to the taste threshold value. The higher the taste activity value is, the greater the contribution of the compound to taste. Mao sums up the content range of taste compounds in Gongfu black tea samples and calculates the taste activity value according to the threshold value. In terms of sourness, L-malic acid, oxalic acid, and aspartic acid are the main sour components of Gongfu black tea, followed by gallic acid and succinic acid. This is different from the research results of Yue et al., which may be caused by different tea varieties [38]. The taste characteristics and threshold values of common organic acids in tea are shown in Table 3.

Some scholars have proved that succinic acid can enhance the umami taste of amino acid compounds [50,51]. Ascorbic acid, citric acid, succinic acid, and malic acid are natural antioxidants that can lower the pH of tea soup, reduce the production of H_2_O_2_, and thus maintain the flavor quality of tea soup [52]. Xu et al. researched the influence of Ca^2+^ on the content of organic acids, turbidity, and sediment formation in green tea soup. The results showed that the turbidity of tea soup is highly negatively correlated with the content of oxalic acid, quinic acid, and tartaric acid [53]. Kaneko et al. are the first to find that theogallin in matcha can enhance the umami taste, and so do succinic acid, and gallic acid [54]. Through correlation analysis, Liu et al. [32] showed that the content of gallic acid and succinic acid in high score group of green tea (characteristic of strong umami taste) is also high and that the total amount of lactic acid, ascorbic acid, and organic acids has a positive effect on the taste of tea soup [55]. Lv et al. analyzed the chemical components of the taste quality of Pu ‘er tea and found that the content level of organic acids was markedly negatively correlated with the score of taste quality [56]. Song et al. showed that fumaric acid had a great contribution to the “mellow and fresh” taste style of black tea. All these results indicate that organic acids play an important role in the taste of tea soup, but the related mechanisms and influencing factors are complicated [57].

## 6. Health Benefits of Organic Acids

The organic acids in tea leaves can promote the human body’s absorption of catechin, enhance the antioxidant properties of tea polyphenols, stimulate the activities of α-amylase and trypsin, and promote the digestion and absorption of starch and protein [40,58,59,60]. In addition, organic acids inhibit the growth of pathogenic intestinal bacteria and improve intestinal function. In the pile-fermentation of Fuzhuan tea, a large number of organic acids are produced, which can reduce the pH value in the intestine, inhibit the growth and reproduction of pathogenic bacteria, and improve the gastrointestinal function of the human body [61,62]. Dark tea extracts containing organic acids and other active ingredients could accelerate gastrointestinal transit, promote colonization of probiotics, including *Bifidobacterium* and *Lactobacillus*, and suppress the proliferation of harmful *Enterococcus* and *Escherichia coli* in normal mice [63]. The antibacterial effect of organic acids in white tea has been studied, and the results showed that all organic acids had inhibitory effects on *Staphylococcus aureus, Shigella flexneri,* and enteric subspecies of *Salmonella enterica*, among which mixed acid had the best antibacterial effect, followed by oxalic acid and acetic acid, and fumaric acid had the worst effect [64].

Citric acid and malic acid are both important products of the tricarboxylic acid cycle. Due to their special roles in metabolism, they can directly participate in metabolic activities in the human body. Malic acid provides energy for the human body for a short period, which can resist fatigue and protect the liver, kidneys, and heart, so it can be used to develop healthy drinks. The addition of malic acid can increase drug stability and promote the absorption and diffusion of the drug in the human body. In addition, L-malic acid has a good antioxidant ability, which can delay the rancidity of food caused by oxidation and the reduction of its nutritional value. Clinically, sodium citrate is used as an anticoagulant, and ferric ammonium citrate is a blood tonic. Citric acid is an important intermediate product of the metabolism of carbohydrates, fat, and protein in animals and plants. In the aerobic state, pyruvate is completely oxidized through the tricarboxylic acid cycle, which is the most efficient way to obtain energy. In addition, salicylic acid, acetoacetic acid, palmitic acid, etc., also have antioxidant, heat-clearing, detoxification, sterilization, and other health benefits.

## 7. Summary and Prospect

Organic acids not only play an important role in the nutrient absorption of tea plants but also contribute to the aroma, taste, and health benefits of tea. So far, it is known that varieties, seasons, environmental conditions, such as light and soil pH, and processing technology affect the composition and content of organic acids in tea plants and different types of finished teas. Compared with other quality and functional ingredients in tea leaves, there are fewer studies on the characteristics of organic acids in tea. This article reviewed the research progresses of organic acids in tea, including their analysis methods, secretion and physiological function of organic acids from tea tree rhizosphere, the composition of organic acids in tea leaves and relevant influencing factors, the contribution of organic acids to sensory quality, and their health benefits, such as antioxidation, promotion of digestion and absorption, acceleration of gastrointestinal transit, and regulation of intestinal flora. The in-depth study of organic acids is conducive to opening up new horizons in the selection and breeding of tea varieties, the optimization of processing technology, and the research on the health functions of tea.

## Figures and Tables

**Table 1:** Analysis methods of organic acids.

Methods	Principle	Characteristics
Titration	Titration end points are determined by indicators or potential changes.	Determination of total, not individual organic acid. Convenient with low sensitivity.
Atomic-absorption spectrophotometry	Oxalate is precipitated into calcium oxalate.	Determination of oxalic acid.
GC-MS	The samples are separated according to the difference in boiling point, polarity, and adsorbability.	Qualitative and quantitative analysis of small organic acid molecules, but not applicable for large molecules.
Ion exchange chromatography	Separation is achieved according to the difference in the ability of the separated components to undergo ion exchange with the stationary phase.	Not suitable for samples with complex composition.
High-performance capillary electrophoresis	Separation is based on the electrophoretic differences between components in a sample.	High-separation efficiency. Less sample loading and relatively poor repeatability.
HPLC-UV	Each component has different partition coefficients in the two phases.	High-separation efficiency. Less sample loading and the use of phosphates, which is easy to form crystals to block the pipeline.

**Table 2:** Determination conditions of major organic acids in tea under MRM-MS.

Organic Acids	Formula	Precursor Ions (m·z^−1^)	Daughter Ion (m·z^−1^)	Cone (V)	Collision (V)
Quinic acid	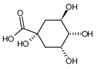	191	85.0 * 93	35	20
Malic acid	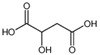	133	114.9 * 45	49	16
Citric acid	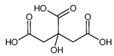	191.2	110.0 * 87	50	18
Succinic acid	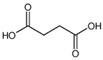	116.9	73.1 * 99.2	60	19
Fumaric Acid	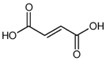	114.9	70.9 * 97.8	68	11.5
Tartaric acid	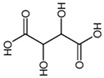	149	102.9 * 73	56.9	15.3
Lactic acid	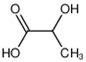	89	43.2 * 70.9	63	21
Gallic acid	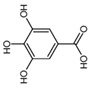	168.9	78.8 *	77	29
Pyruvic acid	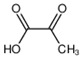	87	43.1 * 68.7	50	12.1
Ascorbic acid	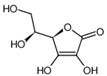	175	87 114.9 *	56	19.1
Shikimic acid	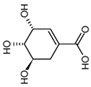	173	93.0 * 136.8	65.4	19.4
Chlorogenic acid	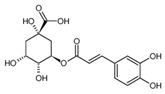	353.1	191.1 *	17	22
Cinnamic acid	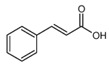	146.9	103.0 *	48	14
Benzoic acid	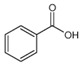	120.9	77.0 *	40	17
Salicylic acid	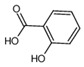	137	92.9 *	48	21

* Represents quantitative ion.

**Table 3:** Taste characteristics and thresholds of common organic acids in tea.

Organic Acid Name	Flavor Characteristics	Threshold Value (mg·L^−1^)
Gallic acid	Sour, astringent	34.02
Oxalic acid	Sour	45
Pyruvic acid	Sour	130
L-malic acid	Sour	87
L-ascorbic acid	Sour	123.28
Lactic acid	Sour	133.2
Acetic acid	Sour	50
Citric acid	Sour	770
Butanedioic acid	Sour	106.28
